# Biomechanical Analysis of Cervical Artificial Disc Replacement Using Cervical Subtotal Discectomy Prosthesis

**DOI:** 10.3389/fbioe.2021.680769

**Published:** 2021-07-14

**Authors:** Jin Wo, Zhenjing Lv, Jing Wang, Kui Shen, Haoran Zhu, Yang Liu, Yuen Huang, Guodong Sun, Zhizhong Li

**Affiliations:** ^1^Department of Orthopedics, First Affiliated Hospital, Jinan University, Guangzhou, China; ^2^Department of Spine Orthopedics, Guangdong Hospital of Integrated Traditional Chinese and Western Medicine, Foshan, China; ^3^Department of Neurosurgery, First Affiliated Hospital, Jinan University, Guangzhou, China; ^4^Department of Rehabilitation, First Affiliated Hospital, Jinan University, Guangzhou, China; ^5^Department of Orthopedics, Fifth Affiliated Hospital, Heyuan Shenhe People's Hospital, Jinan University, Heyuan, China; ^6^Department of Orthopedics, Heyuan People's Hospital, Heyuan Affiliated Hospital of Jinan University, Heyuan, China

**Keywords:** biomechanics, cervical artificial disc replacement, finite element analysis, prosthesis, range of segmental motion, stress, facet joint

## Abstract

**Background:** Anterior cervical discectomy and fusion (ACDF) sacrifices segmental mobility, which can lead to the acceleration of adjacent segment degeneration. The challenge has promoted cervical artificial disc replacement (CADR) as a substitute for ACDF. However, CADR has revealed a series of new issues that are not found in ACDF, such as hypermobility, subsidence, and wear phenomenon. This study designed a cervical subtotal discectomy prosthesis (CSDP) consisting of a cervical disc prosthesis structure (CDP structure), cervical vertebra fixation structure (CVF structure), link structure, and locking screw, aiming to facilitate motion control and reduce subsidence. The aim of this study was to assess the biomechanics of the CSDP using finite element (FE) analysis, friction-wear test, and non-human primates implantation study.

**Study Design:** For the FE analysis, based on an intact FE C_2_-C_7_ spinal model, a CSDP was implanted at C_5_-C_6_ to establish the CSDP FE model and compare it with the Prestige LP prosthesis (Medtronic Sofamor Danek, Minneapolis, MN, United States). The range of motion (ROM), bone-implant interface stress, and facet joint force were calculated under flexion extension, lateral bending, and axial rotation. In addition, CSDP was elevated 1 mm to mimic an improper implantation technique to analyze the biomechanics of CSDP errors in the FE model. Moreover, the friction-wear test was conducted *in vitro* to research CSDP durability and observe surface wear morphology and total wear volume. Finally, the CSDP was implanted into non-human primates, and its properties were evaluated and verified by radiology.

**Results:** In the FE analysis, the ROM of the CSDP FE model was close to that of the intact FE model in the operative and adjacent segments. In the operative segment, the CSDP error FE model increased ROM in flexion extension, lateral bending, and axial rotation. The maximum stress in the CSDP FE model was similar to that of the intact FE model and was located in the peripheral cortical bone region. The facet joint force changes were minimal in extension, lateral bending, and axial rotation loads in CSDP. In the friction-wear test, after the 150-W movement simulation, both the CVF-link-junction and the CDP-link-junction had slight wear. In the CSDP non-human primate implantation study, no subsidence, dislocation, or loosening was observed.

**Conclusion:** In the FE analysis, the biomechanical parameters of the CSDP FE model were relatively close to those of the intact FE model when compared with the Prestige LP FE model. In terms of CSDP error FE models, we demonstrated that the implantation position influences CSDP performance, such as ROM, bone-implant interface stress, and facet joint force. In addition, we performed a friction-wear test on the CSDP to prove its durability. Finally, CSDP studies with non-human primates have shown that the CSDP is effective.

## Introduction

Anterior cervical discectomy and fusion (ACDF) has been successfully applied to obtain functional recovery in degenerative disc disease; however, the treatment requires fusing segments (Mo et al., 2015). Although clinical evidence is still not sufficient to verify that adjacent segment degeneration is caused by the fusion, it is widely recognized that the range of motion (ROM) at non-fused levels will increase inevitably when segmental motion is abolished by the fusion. The increased ROM was considered to be linked with intervertebral disc pressure and even non-fused segment degeneration (Hilibrand and Robbins, 2004; Dmitriev et al., 2005; Carrier et al., 2013). Additionally, a reoperation rate of 10% was caused by other ACDF complications, such as implantation site pain and implant-bone non-union (Zhong et al., 2016). These issues have facilitated the development of cervical artificial disc replacement (CADR) as a substitute method for ACDF.

As an alternative method, CADR preserves segmental mobility by maintaining adjacent intervertebral disc pressure and avoiding adjacent segment degeneration (Sasso et al., 2011; Pandey et al., 2017). To date, the U.S. Food and Drug Administration (FDA) has approved seven CADR devices (Nunley et al., 2018). Most of these devices have polymer-on-metal or metal-on-metal designs to form ball-in-socket sliding articulation (Gandhi et al., 2015). These prostheses have produced satisfactory testing results in clinical trials. However, they also have some problems, such as subsidence, dislocation, and wear phenomenon (Di Martino et al., 2015).

Among these issues, subsidence has been one of the most commonly reported problems, with an incidence of 3–10% (Anderson and Rouleau, 2004). Moreover, reduced bone mineral density caused by overpolishing the end plate and prosthesis design-related uneven stress distribution exacerbate subsidence (Anderson and Rouleau, 2004; Thaler et al., 2013). The wear phenomenon is a physical process caused by motion across a bearing surface. In prostheses, it is associated with the formation of particular wear debris, loss of joint height, and, ultimately, joint failure. More importantly, the particulate debris will induce inflammation mediated by various cytokines. This inflammatory response can lead to pain, osteolysis, and prosthetic loosening (Anderson and Rouleau, 2004; Matge et al., 2015). Additionally, previous research has suggested that the ball-in-socket sliding articulation may induce hypermobility at the surgical level, leading to increased stress on the operative segment and facet joints (Chang et al., 2007b; Kowalczyk et al., 2011; Lee et al., 2011). This stress may play an important role in “operative segment degeneration,” which is one of the major factors that may compromise the long-term results of CADR (Rundell et al., 2008). Thus, the above-mentioned problems have become the focus of CADR improvements and need to be considered when developing new artificial cervical discs.

In this study, we have designed a cervical subtotal discectomy prosthesis (CSDP), consisting of the cervical disc prosthesis structure (CDP structure), cervical vertebra fixation structure (CVF structure), link structure, and locking screw. Artificial disc designs will behave mechanically different because of the distinctiveness of each implant design. These varying designs resulted in different biomechanical alterations in the cervical spine after arthroplasty. Therefore, the purpose of this research was to estimate biomechanical patterns of CSDP at the C_5_-C_6_ level of the cervical spine and to analyze the underlying mechanisms.

The finite element (FE) analysis, an ideal method for research on spine biomechanics, can predict cervical biomechanical responses to different cervical artificial discs (Faizan et al., 2012). Moreover, the FE analysis has unique advantages for measuring biomechanical parameters, such as bone-implant interface stress and implant internal structure stress, which are closely related to subsidence, dislocation, and wear of an implant (Lazaro et al., 2010).

In this experiment, we analyzed and compared biomechanics of the CSDP and Prestige LP prosthesis (Medtronic Sofamor Danek, Minneapolis, MN, United States) by the FE analysis. The main biomechanical parameters included ROM, bone-implant interface stress distribution, and facet joint force. In addition, CADR complications have also been attributed to iatrogenic circumstances, for example, improper positioning of the device (Bertagnoli et al., 2005). Therefore, we moved the CSDP up 1 mm to simulate improper positioning of the device, and the biomechanics were measured by the FE analysis under the same conditions. Moreover, we conducted a friction-wear test *in vitro* to research CSDP durability and to understand the long-term mechanical influences of internal structure interaction. Finally, the CSDP was implanted into non-human primates, and its properties were evaluated and verified by radiology.

## Materials and Methods

### Design Considerations and Material Specifications of CSDP

**Figure 1:**
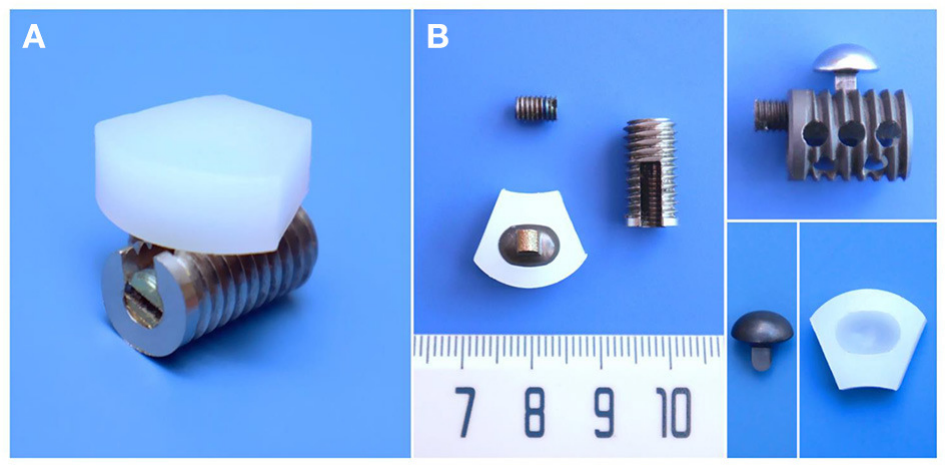
Structural design and material specifications of cervical subtotal discectomy prosthesis (CSDP). **(A)** Oblique views of the assembled CSDP. **(B)** The CSDP consists of cervical disc prosthesis (CDP) structure, cervical vertebra fixation (CVF) structure, link structure, and locking screw. The link structure constitutes the ellipsoid-in-socket articulation with the CDP structure and is fixed on the CVF structure by the locking screw.

The CSDP itself consists of four primary components: CDP structure (ultrahigh molecular weight polyethylene, UHMWPE), CVF structure, link structure, and locking screw (titanium alloy). As an artificial cervical disc, the motion function of the CSDP depends on the articulation composed of the CDP structure and link structure. Different from ball-in-socket articulation-designed artificial discs, the CSDP has an ellipsoid-in-socket articulation design to limit hypermobility. In addition to constituting the articulation of CSDP, another function of the link structure is affixing the CDP structure to the CVF structure by locking screws. The CVF structure is cylindrical with screw threads on the surface. Similar to “hemiarthroplasty,” CSDP fixation depends on the CVF structure in the vertebra; therefore, the CVF structure should be implanted first in CSDP surgery. Before CVF-structure implantation, the inferior vertebra at the operative level was grooved using curettage and a high-speed burr, and the groove was placed close to the upper end plate. Then, the CVF structure was screwed into the groove for early fixation. Moreover, several tunnels, similar to a cervical fusion cage, were reserved in the CVF structure to achieve fusion after long-term implantation (Figure 1).

### Finite Element Biomechanical Analysis

#### Development of Intact FE Cervical Spine Model

**Figure 2:**
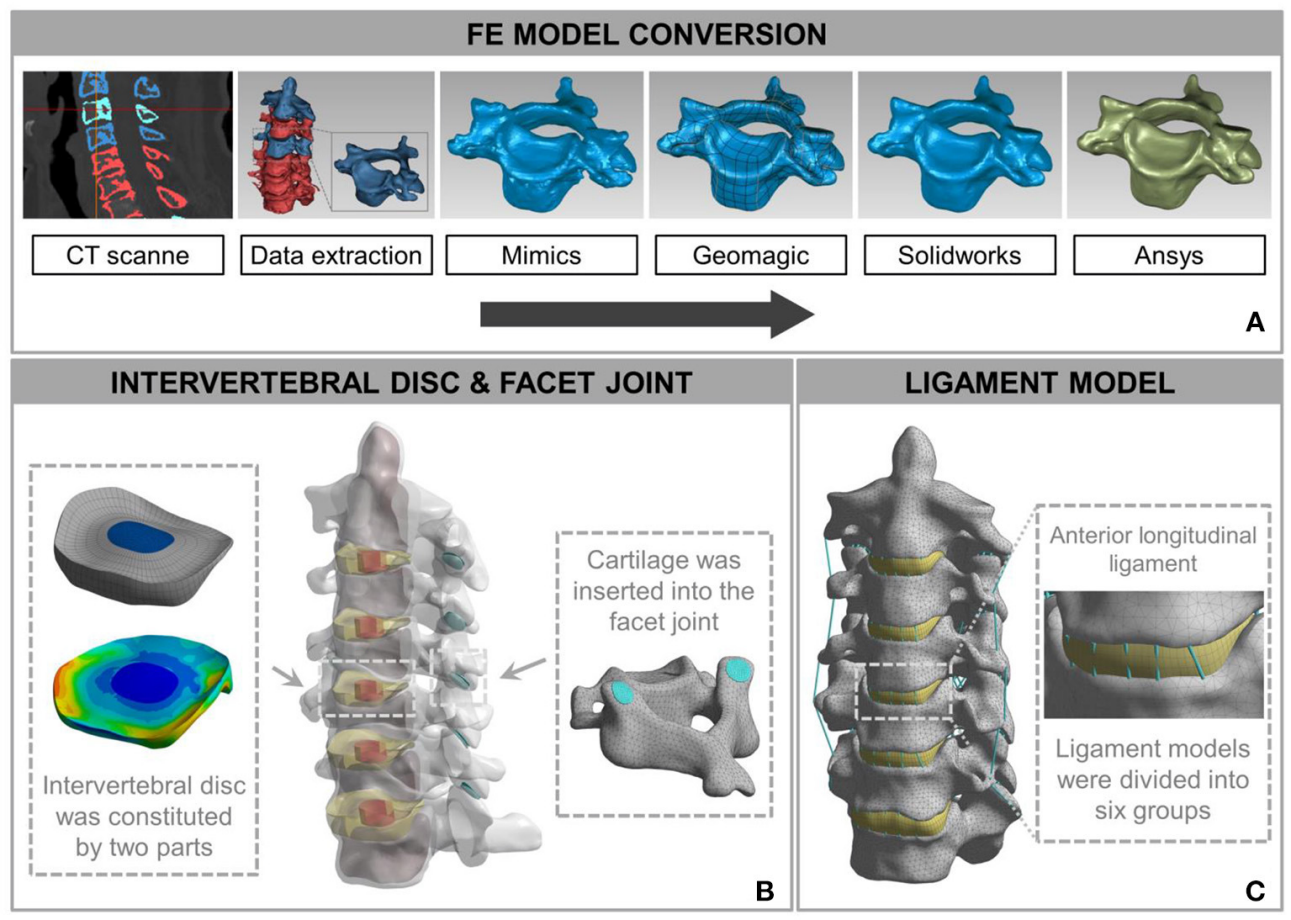
Development and validation of intact finite element (FE) cervical spine model. **(A)** The conversion procedure for developing FE cervical vertebrae models included reconstructing a geometrical structure of vertebrae (Mimics 20.0), performing smooth operation (Geomagic 12), and supporting format conversion by computer-aided design (CAD) software (Solidworks 2015). Then, the output document was imported into FE software (Ansys Workbench 18.0) to build the cervical spine components. **(B)** A model consisting of a vertebra disc and an intervertebral disc was constructed by cartilage and intervertebral discs inserted into the facet joint and the intervertebral space. **(C)** The intact FE cervical spine model with ligament construction.

The FE model of C_2_-C_7_ was developed based on CT images of a healthy subject (male, age 31 years, height 175 cm, weight 74 kg) without radiographic changes in cervical vertebrae or a history of cervical disc disease. The procedure for developing the intact C_2_-C_7_ FE cervical spine model is shown in Figure 2. Computed tomography scans of the subject were obtained at 0.5-mm intervals. Computed tomographic images re-established the three-dimensional structure of the vertebrae by image-processing software (Mimics 20.0, Materialise, Leuven, Belgium) and were then executed according to the smooth operation (Geomagic 12, Geomagic, Morrisville, NC, United States). After format conversion by computer-aided design (CAD) software (Solidworks 2015, Dassault, Vélizy-Villacoublay, France), the output was imported into FE software (Ansys Workbench 18.0, Ansys, Canonsburg, PA, United States) to construct cervical vertebrae (Figure 2A). The cartilages were imported into articular processes to constitute facet joints, and the frictional coefficient was set at 0.1. The intervertebral discs were divided into two parts: nucleus pulposus and annulus fibrosus. The FE model composed of the cervical vertebrae, facet joints, and intervertebral discs was built using the above process (Figure 2B). The ligament models contained the anterior longitudinal ligament, capsular ligament, posterior longitudinal ligament, interspinous ligament, supraspinous ligament, and ligamentum flavum, which were divided into six groups with geometrical linear contact elements utilizing tension.

The calculation time of the three meshes (mesh 1: 0.5 mm; mesh 2: 1 mm; and mesh 3: 1.5 mm) in the same configuration of the same computer were 98, 56, and 24 min. The differences between the tissues of mesh 1 and mesh 2 were <1%. Mesh 2 was considered to be a convergent mesh of intact FE cervical spine model. The numbers of nodes and elements in the intact FE cervical spine model were 446,263 and 226,402, respectively, which guaranteed the accuracy of calculations related to the mesh itself. The model and material properties were set based on previously published literature (Ng et al., 2004; Zhang et al., 2006; Lee et al., 2011; Yu et al., 2016). Material properties of the prostheses and the cervical spine components are presented in Table 1. Finally, we established an FE model of the intact C_2_-C_7_ spinal segment (Figure 2C).

**Table 1:** Material property and mesh type of the prostheses and cervical spine components.

**Component**	**Young modulus (MPa)**	**Poisson ratio**	**Cross section area (mm^**2**^)**	**Element type**	**References**
**Bone**					
Cortical bone	12,000.0	0.29	–	Tetrahedron	Ng et al., 2004; Zhang et al., 2006; Lee et al., 2011
Cancellous bone	450.0	0.29	–	Tetrahedron	
Post bone	3,500.0	0.29	–	Tetrahedron	
End plate	500.0	0.40	–	Tetrahedron	
Cartilage	10.4	0.40	–	Hexahedron	
Nucleus	1.0	0.49	–	Hexahedron	
Annulus	3.4	0.40	–	Hexahedron	
**Ligaments**					
Anterior longitudinal	10.0	0.30	6.0	Link	Lee et al., 2011; Yu et al., 2016
Posterior longitudinal	10.0	0.30	5.0	Link	
Ligamentumflavum	1.5	0.30	5.0	Link	
Interspinous	1.5	0.30	10.0	Link	
Supraspinous	1.5	0.30	5.0	Link	
Capsular	10.0	0.30	46.0	Link	
**Artificial disc**					
Titanium alloy	110,000.0	0.30	–	Tetrahedron	Lee et al., 2011; Yu et al., 2016
UHMWPE	3,000.0	0.30	–	Tetrahedron	

For validation, the intact FE model was loaded in flexion extension, lateral bending, and axial rotation by imposing 1.5 Nm on C_2_ with C_7_ firmly fixed. For this purpose, on the middle top of C2–C7, six distinctive measuring material points were identified. The angles that were produced by the vector connected by adjacent points before and after the simulation depicted the ROM from C2 to C7. Under respective loading situations in light of prior experiments, ROM was compared with outcomes in the literature by Pelker et al. (1991), Panjabi et al. (2001), Kubo et al. (2003) and Ng et al. (2004) aiming to evaluate the validity of the intact FE model.

#### Development of CADR FE Model

**Figure 3:**
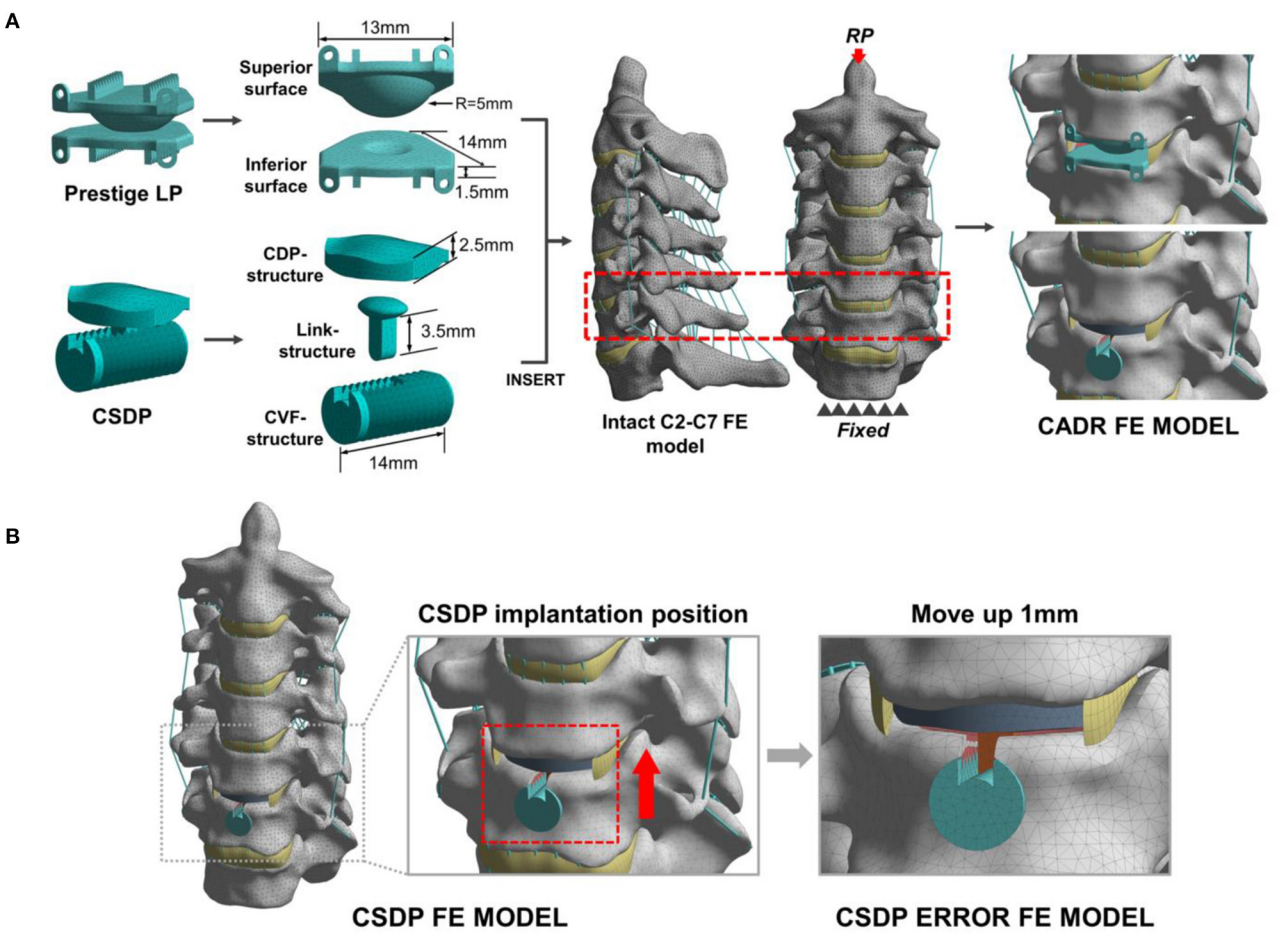
Development and experimental conditions of the cervical artificial disc replacement (CADR) FE model. **(A)** The Prestige LP finite element (FE) model was composed of superior surface and inferior surface structures. The cervical subtotal discectomy prosthesis (CSDP) FE model was divided into three parts: cervical disc prosthesis (CDP) structure, cervical vertebra fixation (CVF) structure, and link structure. The Prestige LP and CSDP FE models were implanted at C_5_-C_6_. **(B)** The CSDP was moved up by 1 mm to simulate an imprecise surgical insertion situation as a CSDP error FE model.

The CSDP and Prestige LP were modeled using actual specimen sizes and material properties available in the literature (Table 2). The Prestige LP model consisted of two titanium end plates with the upside-down dome of the superior end plate articulating with the groove of the inferior end plate, and with the frictional coefficient set at 0.2. In order to simplify the CSDP model, three structures were constructed, namely, the CDP structure, CVF structure, and link structure. The CDP structure was made of UHMWPE, and the CVF structure and link structure were made entirely of titanium alloy. The CVF structure and link structure were set to bond upon contact, replacing the function of the locking screw. Friction contact was also used for the ellipsoid-in-socket articulation of CSDP constituted by the CDP structure and link structure, and the frictional coefficient was set at 0.08 (Figure 3A).

**Table 2:** ROM validation of intact FE cervical spine model.

**Segment**	**Flexion extension (ROM)**		**Lateral bending (ROM)**		**Axial rotation (ROM)**	
	**Intact FE model (^**°**^)**	**Range (^**°**^)**	**Intact FE model (^**°**^)**	**Range (^**°**^)**	**Intact FE model (^**°**^)**	**Range (^**°**^)**
C_2_/C_3_	6.3	5.9–7.5	4.9	3.4–15.4	5.6	2.3–7.7
C_3_/C_4_	7.9	7.3–11.5	4.5	3.4–15.4	7.8	2.3–13.0
C_4_/C_5_	8.0	7.4–10.1	4.2	3.4–15.4	7.8	2.3–13.6
C_5_/C_6_	8.4	7.2–9.9	3.7	3.1–15.4	5.9	2.3–13.8
C_6_/C_7_	7.9	5.7–11.5	3.7	3.4–15.4	4.8	2.1–10.8

The two models were implanted into the C_5_-C_6_ segment, where CADR is most frequently carried out. To imitate the Prestige LP insertion, the C_5_-C_6_ anterior longitudinal ligament, intervertebral disc, and end plate were removed. Then, the Prestige LP model was implanted at the C_5_-C_6_ segment in accordance with the clinical condition. During the CSDP insertion process, first, the CVF structure was inserted, and the cylindrical bone of the C_6_ vertebra was removed. Following this procedure, the CDP structure was implanted, after which the anterior longitudinal ligaments, nucleus pulposus, annulus fibrosus, and 20% of the C_6_ end plate were removed without removing the C_5_ end plate. In addition, we moved the CSDP up by 1 mm to mimic an imprecise surgical insertion situation as a CSDP error FE model (Figure 3B). The bond upon contact condition was defined at the bone-implant interface.

By applying 1 Nm of flexion extension, lateral bending, and axial torsion combined with a 73.6 N compressive follower load on C_2_, the intact FE, CSDP FE, Prestige LP FE, and CSDP error FE models will bend or rotate under load (Yu et al., 2016). Simultaneously, C_7_ is fixed throughout the loading process. Range of motion, bone-implant interface stress distribution, and facet joint force analysis were carried out by quasistatic testing under the load conditions mentioned above to predict biomechanical patterns at the C_5_-C_6_ level of the cervical spine.

### Friction-Wear Test

The friction-wear test detects wear on the two junctions of CSDP: the junction composed of the CVF structure and the link structure (CVF-link-junction) and the junction composed of the CDP structure and the link structure (CDP-link-junction). The experiment simulates the wear process of the two CSDP junctions after 150-W movements in simulated body fluid (SBF) and non-SBF environments, respectively. The contact stress on CVF-link-junction was 10 MPa and on CDP-link-junction was 5 MPa. The surface wear morphology and total wear volume were measured by the Multi-Function Tribometer (MFT-5000, Rtec, San Jose, CA, United States).

### CSDP Implantation in Non-human Primates

Care and experimental procedures for non-human primates were approved by the Institutional Animal Care and Use Committee (IACUC). This study was conducted in compliance with relevant Chinese law and regulations on the management of laboratory animals promulgated by the State Science and Technology Commission. Eight male *Macaca fascicularis* (Huazhen Biotechnology, Guangzhou, China), 9.2–12.1 years and 9.5–10.2 kg, were fed in an indoor facility accredited by the Association for Assessment and Accreditation of Laboratory Animal Care International. The animals were housed in individual stainless-steel cages in a specific room where an environmental temperature of 21–25°C and a relative humidity range of 40–60% were maintained. Although individually housed, the animals were provided continuous auditory, visual, and olfactory contact with neighboring conspecifics. In addition to the standard non-human primate diet, water and fresh fruits were available *ad libitum*. Small amounts of primate treat and various cage-enrichment devices were supplied.

**Figure 4:**
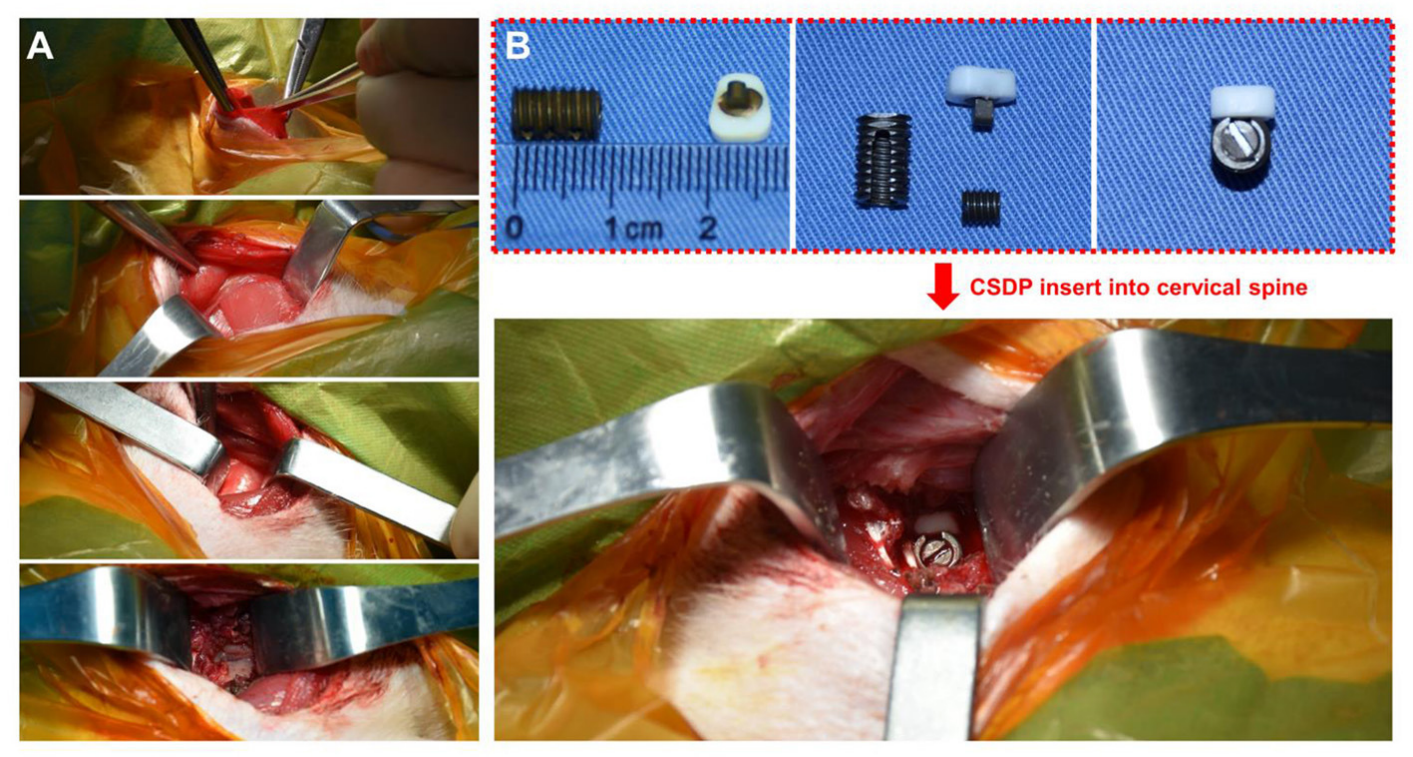
Non-human primate cervical subtotal discectomy prosthesis (CSDP) implantation surgery. **(A)** After separating the skin, the platysma was cut with an electric knife. Then, the envelope fascia was sharply separated until the sternocleidomastoid muscle was seen. The sternocleidomastoid muscle was separated from the scapulohyoid muscle. Finally, the vertebral body was exposed by peeling off the longuscolli. **(B)** The size of the CSDP was modified based on the cervical spine anatomy of the non-human primates before implantation.

Prior to the surgery, each animal was sedated with ketamine (6 mg/kg) followed by endotracheal intubation and general anesthesia using 1.5% isoflurane. The neck area was shaved with razors and prepared with iodophor. The surgery was performed using an aseptic technique. The anterior approach to the cervical spine was adapted to the non-human primate model through a right-sided longitudinal incision (Figure 4A). Once the anterior cervical vertebral elements were exposed, the C_5_-C_6_ intervertebral disc was identified by x-ray, and a CSDP implantation surgery was performed. First, the C_6_ vertebral body was grooved using curettage and a high-speed burr with the groove positioned close to the C_6_ upper end plate. The CVF structure was then implanted at the C_6_ groove. Second, the cartilage of the vertebral end plates was preserved, and the annulus and nucleus pulposus were removed. Finally, the CDP structure and link structure were implanted at C_5_-C_6_ and were fixed on the CVF structure by the lock screw (Figure 4B). The incision was sutured using layers, and the animals were returned to their home cages after recovery from anesthesia.

To prevent postoperative infection, the animals were treated with cefotaxime sodium (50 mg/kg IM, twice a day for 3 days). To alleviate acute postoperative pain, the animals were treated with rotundine (3 mg/kg IM, two times a day for 3 days). The observation was carried out using a CT scanner (Siemens, Munich, Germany) and a 2.0 MRI scanner (Siemens, Munich, Germany) 1 month before surgery and 1 year after surgery.

## Results

### Validation of Intact FE Cervical Spine Model

**Figure 5:**
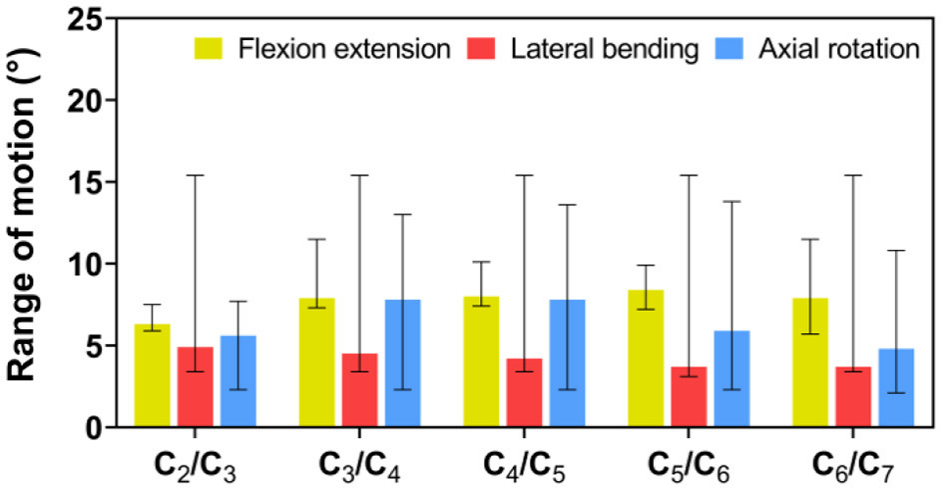
Validation of intact finite element (FE) cervical spine model. Range of motion (ROM) outputs obtained from the intact FE model were compared with the literature data to assess the validity of the model. ROM at each segment in the intact FE model was entirely in the range of literature results.

In the FE analysis, ROM outputs acquired from the intact FE model that we constructed were compared with data from previous experiments to estimate the validity of the model (Penning, 1978; Panjabi et al., 1986, 2001; Penning and Wilmink, 1987; Moroney et al., 1988; Mimura et al., 1989; Pelker et al., 1991; Holmes et al., 1994; Lai et al., 1994; Clausen et al., 1997; Kubo et al., 2003). Range of motion at each segment in the model was all in the range of results observed in previous experimental studies, although the segmental ROM for lateral bending was near the lower bound of the range given in previous experimental studies (Figure 5 and Table 2). Based on these results, we demonstrated the validity of the intact FE cervical spine model.

### Range of Motion of Intact FE Cervical Spine Model and CADR FE Models

#### Flexion–Extension Load

Under the follower load of 73.6 N and the flexion–extension load of 1 Nm, ROM in CSDP and Prestige LP FE models was 28.89° and 31.84°, respectively. Compared with 29.59° in the intact FE model, ROM was decreased by 2.37% in the CSDP FE model and increased by 7.6% in the Prestige LP FE model. Although ROM in flexion extension at the C_5_-C_6_ segment increased by 17.76% in the case of the Prestige LP FE model, the CSDP FE model showed a decrease of 6.57% when contrasted with the intact FE model. The ratio of C_5_-C_6_ and C_2_-C_7_ ROM was 18.21% for the CSDP model and 20.82% for the Prestige LP FE model.

#### Lateral Bending Load

As in the flexion–extension load, no significant differences were found in the intact segments between the intact FE model and the CADR model in the lateral bending load. However, ROM of the C_5_-C_6_ level was 21.4% higher in the Prestige LP FE model and 0.6% lower in CSDP FE model compared with the intact FE model. The ratio of the C_5_-C_6_ ROM with respect to C_2_-C_7_ in the Prestige LP FE model and CSDP FE model was 22.52 and 19.96%, respectively.

#### Axial Rotation Load

**Figure 6:**
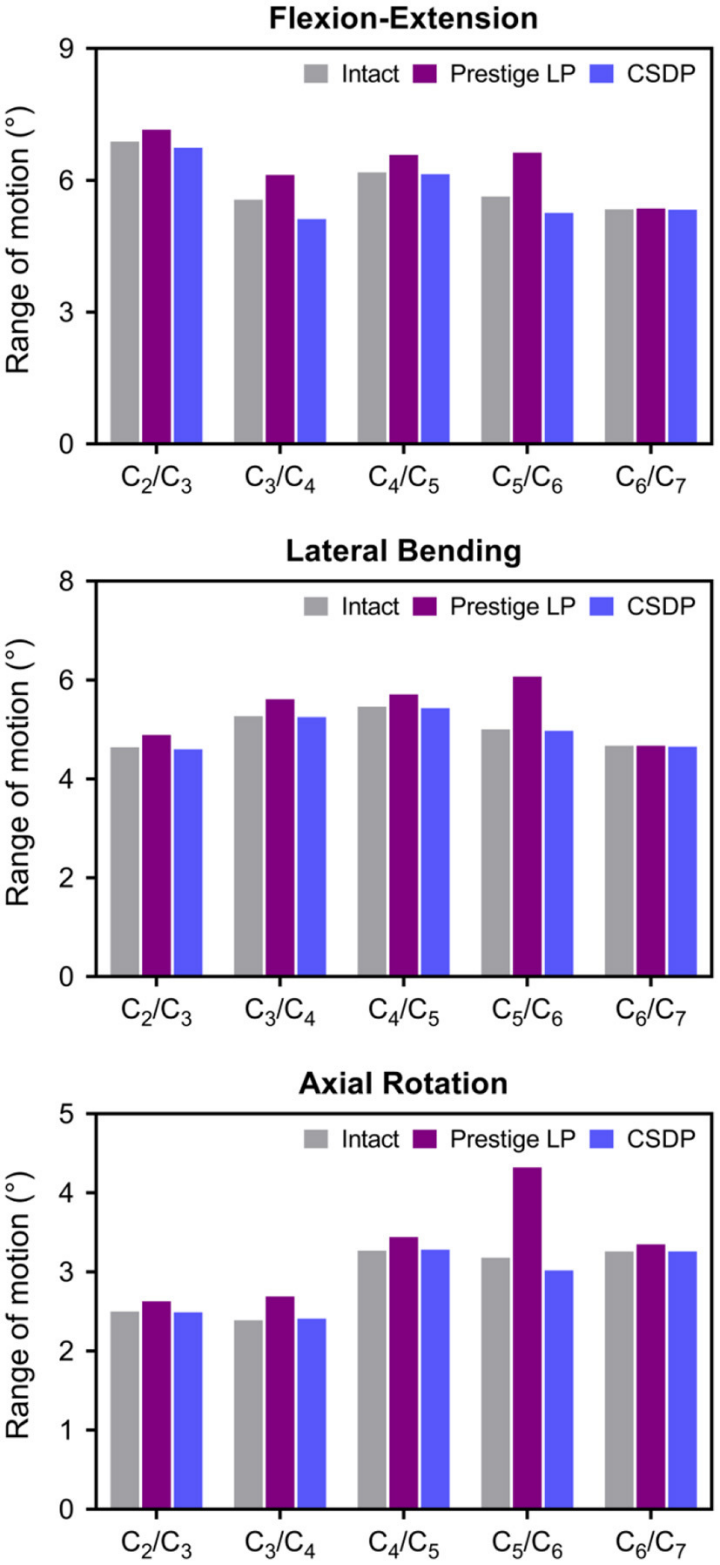
Range of motion (ROM) of intact finite element (FE) cervical spine model and cervical artificial disc replacement (CADR) FE models. In three loads, no significant differences were found at the C_5_-C_6_ level and other segments between the intact FE and cervical subtotal discectomy prosthesis (CSDP) FE models. ROM at the C_5_-C_6_ level was higher in the Prestige LP FE model than in the intact and CSDP FE models.

Under axial rotation load, no significant differences were found in the intact segments between the intact FE and CADR models. Compared with 14.6° in the intact FE model, ROM in the Prestige LP FE model increased by 12.53% and decreased by 0.96% in the CSDP FE model. Range of motion at the C_5_-C_6_ level was 35.85% higher in the Prestige LP FE model and 5.03% lower in the CSDP FE model when compared with the intact FE model. The ratio of the C_5_-C_6_ ROM with respect to C_2_-C_7_ in the Prestige LP and CSDP FE models was 26.29 and 20.89%, respectively, and differed from the 21.78% in the intact FE model (Figure 6).

### Stress Analysis of Intact FE Cervical Spine Model and CADR FE Models

#### Von Mises Stress

**Figure 7:**
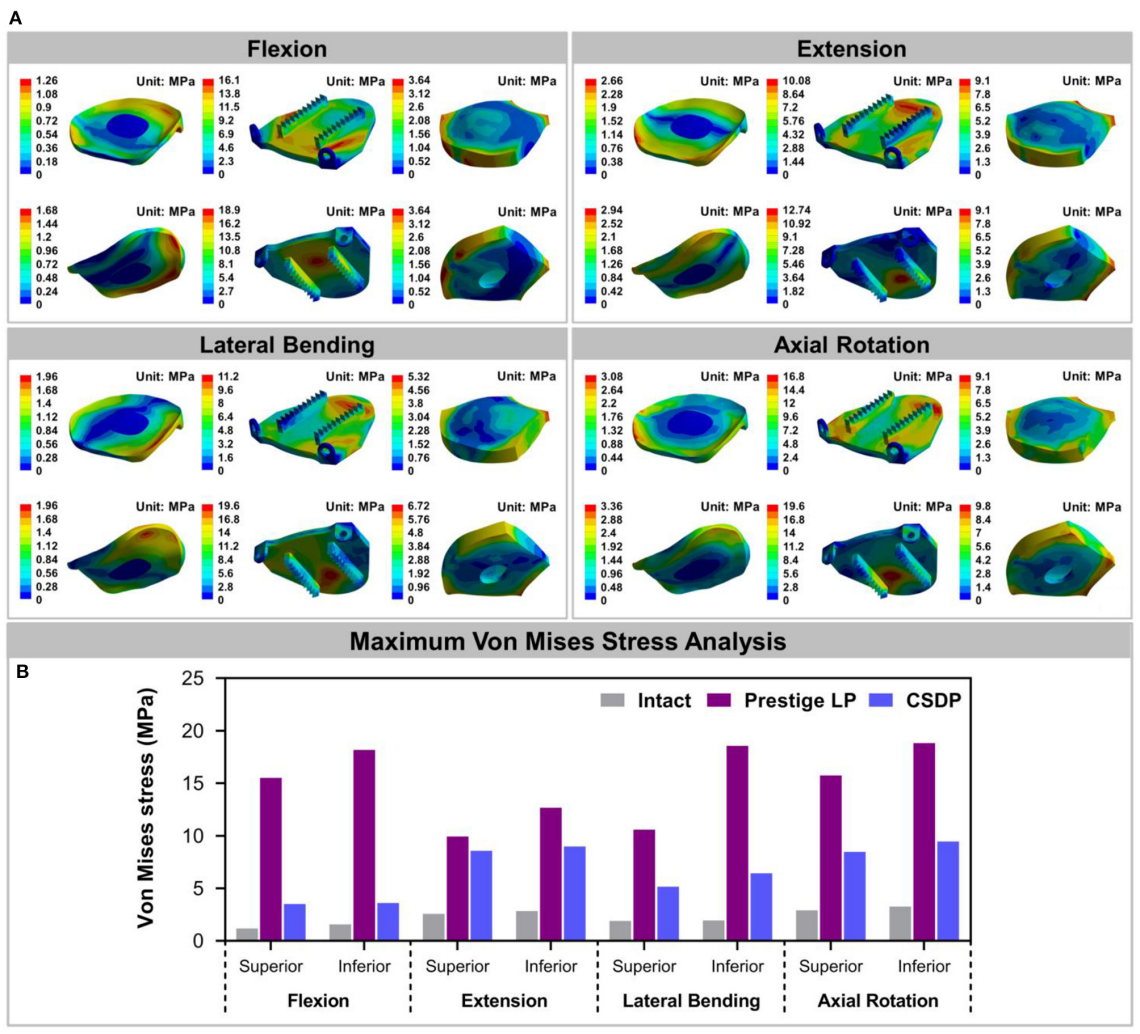
Stress analysis of the intact finite element (FE) cervical spine model and cervical artificial disc replacement (CADR) FE models. **(A)** The Von Mises stress can be observed, including intact, Prestige LP, and cervical subtotal discectomy prosthesis–cervical disc prosthesis (CSDP-CDP) structure FE models in flexion, extension, lateral bending, and axial rotation loads. Stress of the Prestige LP FE model, distributed in the central region, was much higher than that of the CDP structure FE and intact FE models. Stress distribution of the CSDP-CDP structure FE model was similar to that of the intact FE model, located in the peripheral region. **(B)** Maximum Von Mises stress analysis of the intact, Prestige LP, and CSDP-CDP structure FE models.

The Von Mises stress on the bone-implant interface in CADR FE models in flexion, extension, lateral bending, and axial rotation is shown in Figure 7A. Maximum stress on the inferior surface in the intact, Prestige LP, and CSDP-CDP-structure FE models was higher than that on the superior surface of these models (Figure 7B). Stress was concentrated in the central region in the Prestige LP FE model, and the average stress was much higher than in the CSDP-CDP structure and intact FE models. The maximum stress in the Prestige LP FE model was 18.839 MPa, observed in axial rotation loading. In addition, maximum stress was 3.267 and 9.464 MPa in the intact and CSDP-CDP structure FE models, respectively. The stress distribution of the CSDP-CDP structure FE model showed a trend similar to that of the intact FE model, which was located in the peripheral region but had relatively higher stress than in the intact FE model.

**Figure 8:**
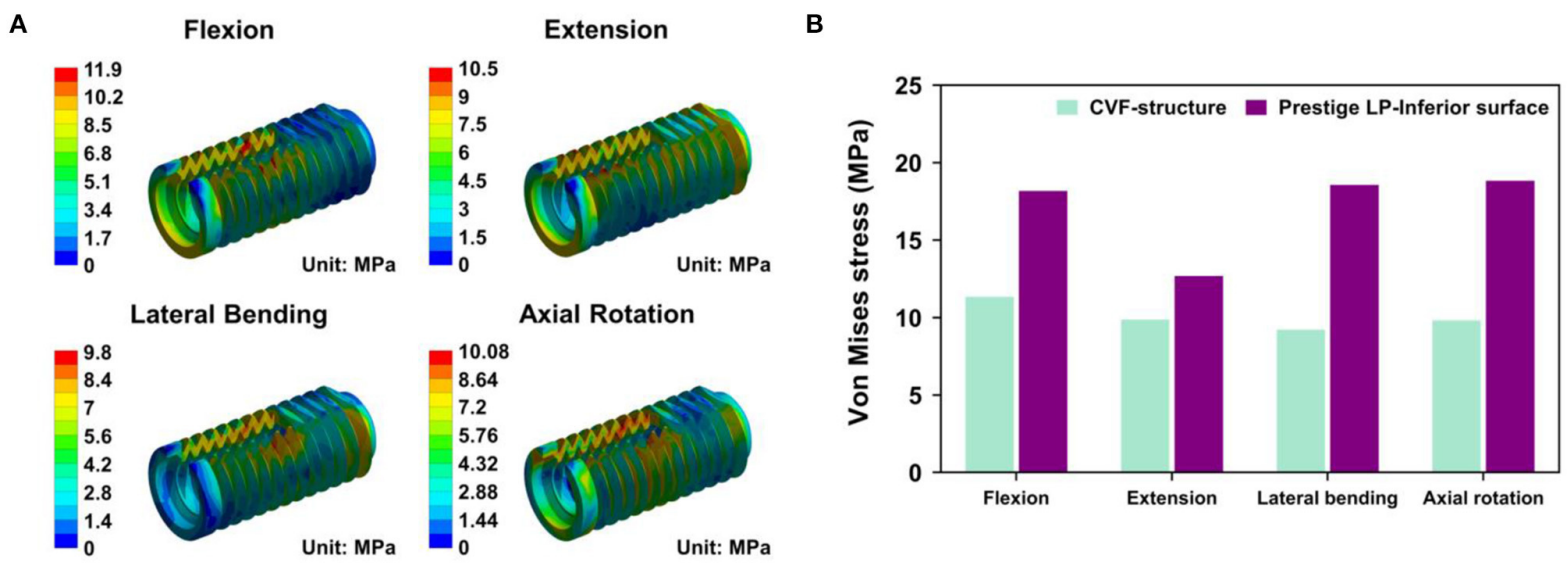
Stress analysis of cervical subtotal discectomy prosthesis–cervical vertebra fixation (CSDP-CVF) structure. **(A)** Maximum stress for the CSDP-CVF structure finite element (FE) model, located nearby the link structure, instead of the bottom. **(B)** Maximum Von Mises stress analysis of CSDP-CVF structure FE models. Maximum stress in the CVF structure was less than that of the Prestige LP inferior surface in all loads.

For the CSDP-CVF structure, maximum stress observed in flexion loading was 11.351 MPa and was nearer to the location of the link structure, instead of the bottom; however, in flexion, the corresponding maximum stress was 18.174 MPa for the Prestige LP FE model (Figure 8).

#### Facet Joint Force

**Figure 9:**
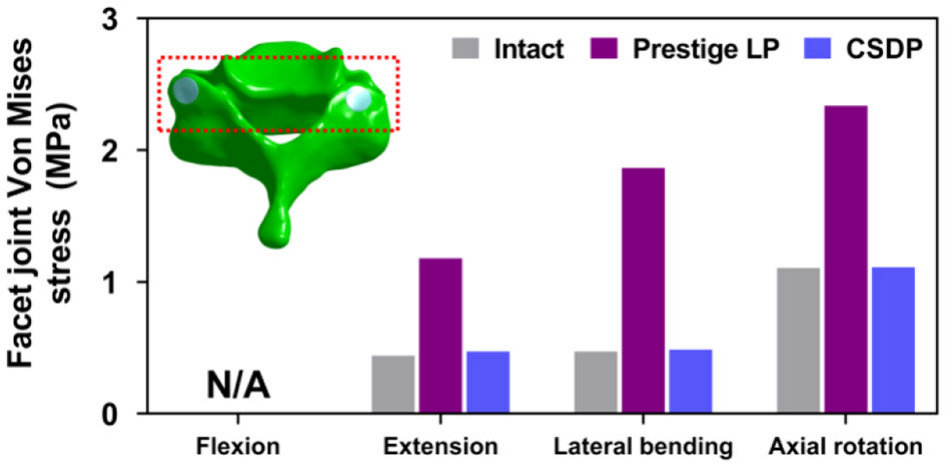
Facet joint force analysis of the cervical subtotal discectomy prosthesis (CSDP) finite element (FE) model.

The outputs of facet joint forces are presented in Figure 9. Under the flexion load, the facet joint was in an extended position, and the pressure value was not measured. In extension loading, the facet joint force increased by 167.95% in the Prestige LP relative to the intact FE model, while the value did not increase extremely in the CSDP FE model. The facet joints force in the lateral bending load within all CADR FE models was higher than in the intact FE model. The variation of lateral bending facet joint force in the Prestige LP and CSDP FE models was 295.13 and 2.86% of the intact value, respectively. Contrasted with the intact FE model in axial rotation, the maximum increase in facet force was 111.35% with the Prestige LP, whereas it was 0.47% with the CSDP FE model.

### Biomechanical Analysis of CSDP Error FE Model

#### Range of Motion of CSDP Error FE Model

**Figure 10:**
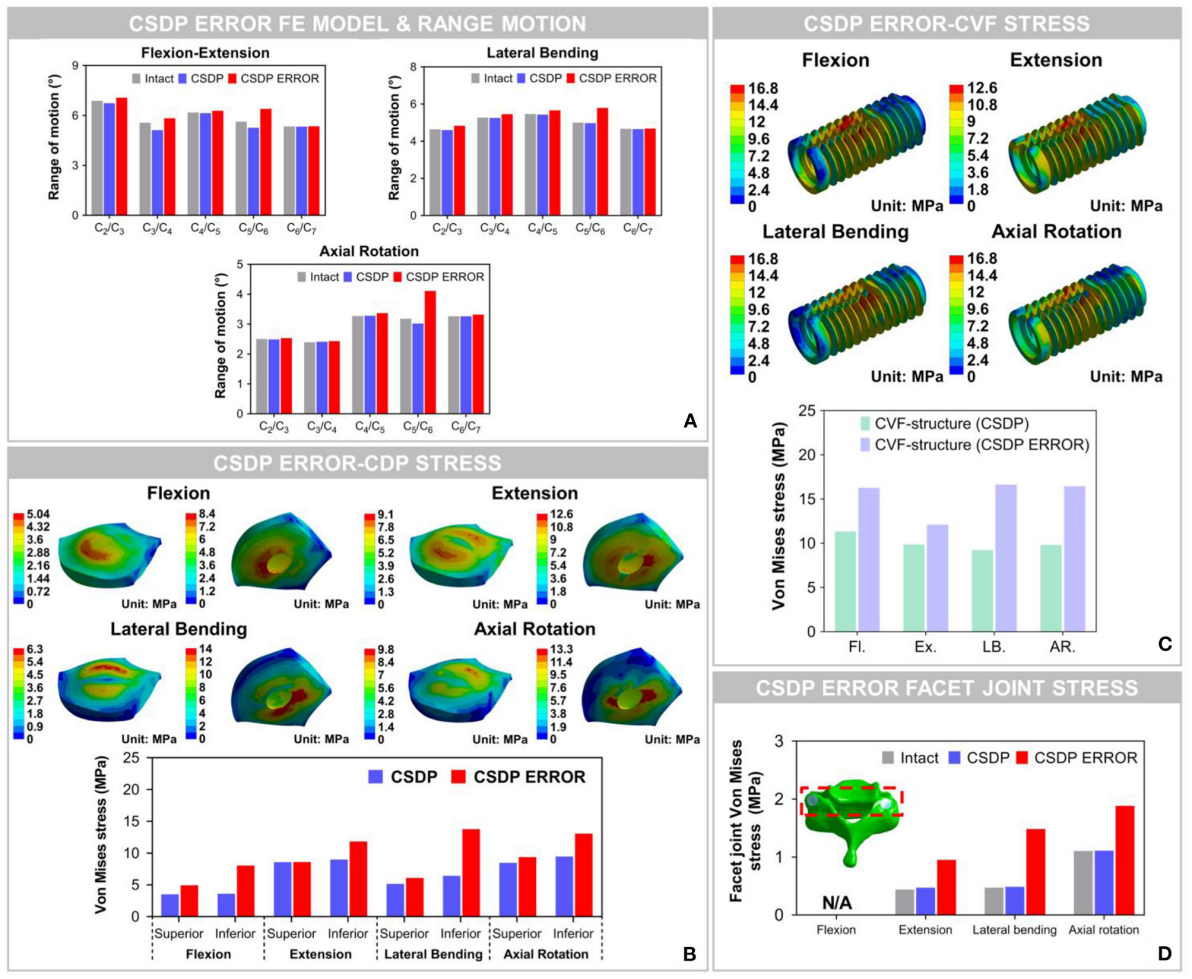
Biomechanical analysis of the cervical subtotal discectomy prosthesis (CSDP) error finite element (FE) model. **(A)** The results illustrated that range of motion (ROM) increased at the C_5_-C_6_ level with CSDP error FE model replacement, and the CSDP error FE model had a greater effect on ROM in axial rotation than in flexion extension and lateral bending. **(B)** After the CSDP FE model was replaced with the CSDP error model, stress was concentrated in the central region of the CSDP error-CDP-structure FE model. **(C)** Stress sustained by the CSDP error-CVF-structure FE model was higher than that by the CSDP-CVF-structure FE model, yet still similar to that by the CSDP FE model. **(D)** Facet joint force within the CSDP error FE model was higher than that in the intact and CSDP FE models. Maximum facet joint force in the CSDP error FE model was observed during axial rotation.

The results showed that ROM at C_2_-C_7_ increased by replacement with the CSDP error FE model, relative to the CSDP FE model. The CSDP error FE model had a significant influence on ROM in axial rotation but not in flexion extension and lateral bending. At the operative segment, with respect to the CSDP FE model, the CSDP error FE model produced a small increase of 21.67 and 16.5% ROM in flexion extension and lateral bending, respectively, while there was a 36.09% increase in axial rotation. Nevertheless, the Prestige LP FE model was more affected than the CSDP error FE model in ROM. When the CSDP error FE model was compared with the Prestige LP FE model, small decreases of 3.59, 4.84, and 5.11% were observed in flexion extension, lateral bending, and axial rotation, respectively (Figure 10A).

#### Von Mises Stress of CSDP Error FE Model

The Von Mises stress on the bone-implant interface of the CSDP error FE model was significantly greater than that in the CSDP FE model. Maximum stress was 9.464 MPa in the CSDP FE model, while in lateral bending it had a value of 13.057 MPa in the CSDP error FE model. Different from the CSDP FE model, the stress distribution of the CSDP error-CDP-structure FE model was observed in the CDP-structure central region and was significantly higher (Figure 10B). The stress sustained by the CSDP error-CVF-structure FE model was still found at the link structure, with the maximum being 16.631 MPa (Figure 10C).

#### Facet Joint Force of CSDP Error FE Model

The maximum stresses on facet joints in the CSDP error FE model were observed during axial rotation. Moreover, in the FE models of intact and CSDP, stress was also observed in axial rotation (Figure 10D). Although the CSDP error FE model produced higher facet-joint force than the CSDP did, the maximum facet joint force in the Prestige LP FE model was greater in all CADR FE models.

### Friction-Wear Test

**Figure 11:**
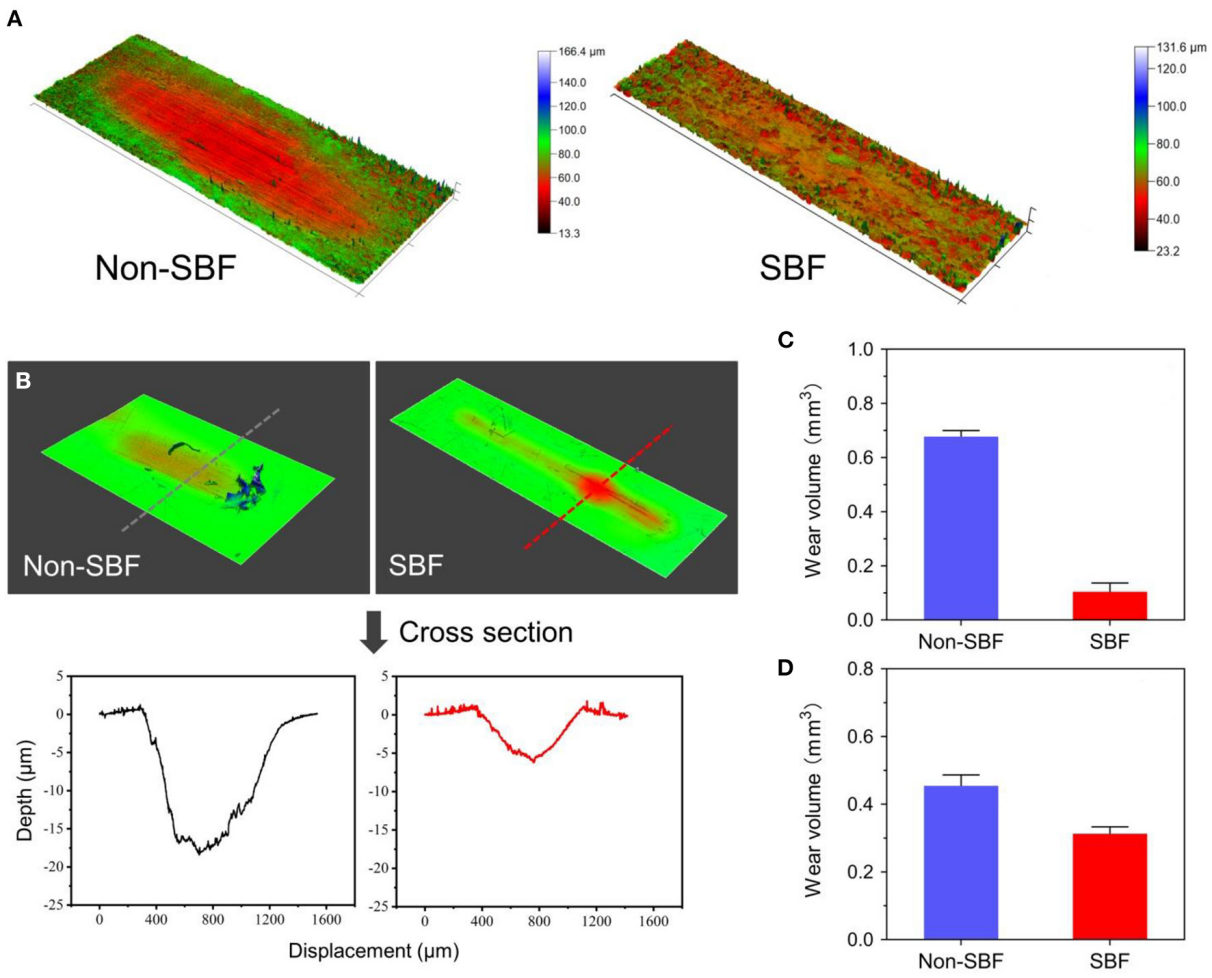
Surface wear morphology observation and wear volume. **(A)** Surface wear morphology observation of cervical vertebra fixation (CVF) link junction; the color represents the degree of wear. **(B)** The wear morphology cross-section of cervical disc prosthesis (CDP) link junction. Total wear volume of the **(C)** CVF link junction and the **(D)** CDP link junction was quantified.

The results of CVF-link-junction and CDP-link-junction in the SBF and non-SBF environments after 150-W movement simulation are shown in Figure 11. In SBF, the CVF-link-junction and CDP-link-junction have slight wear. Especially in the CVF-link-junction, the total wear volume is much lower in SBF than in non-SBF. For the wear morphology cross-section observation of the CDP-link-junction, the wear depth was only approximately 5 μm in the SBF, which is lower than the 20 μm in the non-SBF.

### Radiological Observation of CSDP in Non-human Primates

**Figure 12:**
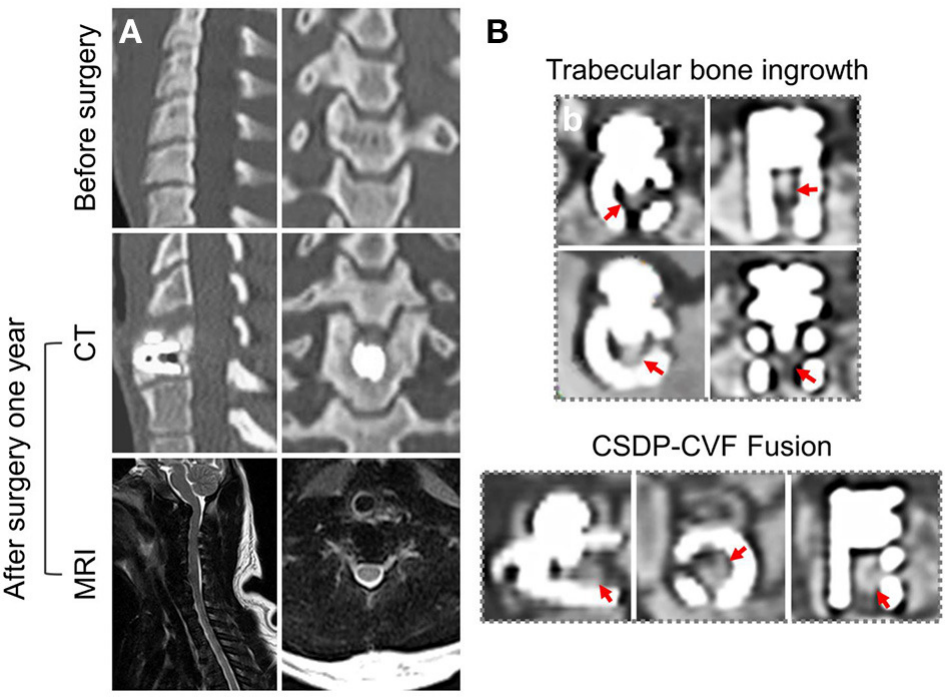
Radiological observation of cervical subtotal discectomy prosthesis (CSDP) in non-human primates. **(A)** CT and MRI 1 month before and 1 year after CSDP implantation in non-human primates. **(B)** The trabecular bone grows into the interior of the cervical vertebra fixation (CVF) structure through the tunnel.

Figure 12 shows the CT and MRI scans 1 month before and 1 year after CSDP implantation in non-human primates. CT showed that after 1 year, CSDP subsidence, dislocation, and loosening were not observed. In addition, 1 year after CSDP implantation, the inside of the CVF structure was filled with the trabecular bone, and the CVF structure had undergone intravertebral fusion. Based on the MRI result, no spinal cord edema, degeneration of the adjacent intervertebral disc, or inflammation of the surrounding vertebral body was observed in the surgical segment.

## Discussion

Cervical artificial disc replacement aims to prevent adjacent segment degeneration by restoring intervertebral disc mobility in degenerative segmental motion. The Prestige LP prosthesis was chosen because of its current global popularity and because it is similar to most ball-in-socket sliding articulations used today (Choi et al., 2020). The Prestige LP is an open two-piece, semi-constrained design with metal-on-metal ball-in-socket articulation. The CSDP is an open four-piece, semi-constrained design with polymer-on-metal ellipsoid-in-socket articulation. Biomechanical studies have shown that ball-in-socket sliding articulation may not substantially control motion and may cause hypermobility at the surgical level (Kowalczyk et al., 2011). Hypermobility was a direct negative factor that increases strain in implanted segments and facet joints. Under the hypermobility condition, increasing the load through the capsular ligament during physiological situations and CADR sliding articulation configuration would alter the load transmit mode at the surgical segment. In this study, ROM distribution through C_2_-C_7_ segments in the CSDP FE model was almost similar to that in the intact FE model, whereas it had changed in the Prestige LP FE model. Although spinal motion in the implanted site was preserved in the Prestige LP FE model, ROM increased by 17–35% compared with the intact FE model, possibly because of a hypermobility condition. In previous studies, similar results have suggested that a significantly increased ROM at the operative segment was found after replacement with the Prestige model (Chang et al., 2007b). The coincidental result of this study and previous *in vivo* research confirmed this conclusion. As for the CSDP error FE model, C_5_-C_6_ ROM was significantly greater than that in the intact and CSDP FE models regardless of motion loads. However, the Prestige LP model generated a greater increase in C_5_-C_6_ ROM than the CSDP error FE model did in both groups.

Subsidence and dislocation are problems that may result from intrinsic design flaws of the devices. The subsidence tendency is associated with interfacial stress increases, leading to a high bone-implant interface stress situation (Lin et al., 2009). Therefore, bone-implant interface stress may dissipate evenly in prostheses rather than in concentrated areas (Anderson and Rouleau, 2004). The stress distribution of the Prestige LP model was uneven and mostly focused on central and posterior regions; the CSDP-CDP structure FE model was similar to the intact FE model located in the peripheral cortical bone region. Moreover, the maximum stress on the superior and inferior surfaces of the Prestige LP FE model was higher compared with the CSDP FE model. It is generally acknowledged that subsidence is most often caused by improper device design that affects end plate preparation and stress distribution; however, a decrease in bone quality can also lead to subsidence (Bertagnoli et al., 2005). Bone resection may affect the structural integrity of end plates, resulting in decreased end plate bone quality. Because of the structural design of the CSDP, the C_5_ end plate could be saved and, therefore, decrease the risk of subsidence during operation.

The anchorage structure of the prosthesis also determines the propensity for subsidence. The potential of an artificial disc to generate interface resorption and subsequent subsidence depends on a variety of biomechanical factors that can be expressed in terms of relative movement between bone and implant at the interface (Weinans et al., 1993). Stress distribution on the surface of the anchorage structure may reflect trends in load transfer and relative movement. Similar to biomechanical disruption of the bone-implant interface of an acetabular cup in total hip replacement, micromotion can also be intensified with the displacement of the anchorage structure relative to vertebral bodies during repetitive loading. The results of stress analysis showed that the CSDP-CVF structure dissipated stress more evenly to provide physiological bonding at the bone-implant interface. With the Prestige LP FE model, high bone-implant interface stress occurred at the posterior flanges on the inferior surface, producing maximum stress at 18.839 MPa.

Device wear and deterioration can occur at any interface, most commonly at the bearing surfaces but also at the host-implant or implant-implant interfaces. Wear production varies, depending on the material used and mechanisms of biomechanical stress applied to the implant. The anchorage structure is indispensable in preventing the migration of the prosthesis; however, stress located in connection with various CSDP structures, especially at the junction of the link structure and CVF structure, is high. In the FE analysis, the maximum stress was observed at the CVF-link-junction. Although the link structure is attached to the CVF structure by a locking screw, it still allows micro-movements. It has been reported that high-stress distribution may increase the risk of wear (Lee et al., 2016). In addition, the movement of the CSDP depends on the joint CDP structure and link structure, which will also cause wear phenomena in the long term. Therefore, we carried out a friction-wear test on these joint structures. In the SBF environment, the surface wear morphology and wear volume of these joint structures illustrated that CSDP has a long-term life. Conversely, the FE analysis showed that the stress on joint interfaces increased in the CSDP error model. Although the stress in the CSDP error-CDP structure FE model was less than the corresponding yield stress of UHMWPE (28 MPa), the stress distribution of the CSDP error-CDP structure, to some extent, increases the risk of wear, which may affect long-term follow-up results. It has been reported that high-stress distribution in the UHMWPE zone may increase the risk of wear inside the core (Lee et al., 2011, 2016). Therefore, the implantation technology of CSDP is vital, especially the implantation position, which can reduce the wear in the CSDP.

Increased force on facet joints after ADR has been cited as a reason for degenerative changes in implanted segments and poor clinical results; however, biomechanical or clinical evidence has not been clear (Huang et al., 2004; Anderson et al., 2008). In this study, the stress sustained by facet joints increased by 7.3% in the case of the CSDP FE model, and by 167.9 and 115.9% in the case of the Prestige LP and CSDP error models, respectively, demonstrating a remarkable stress increase in CADR segments. Chang et al. (2007a) reported that stress increased by 25.1% under an extension load in comparison with intact segments. Rundell et al. (2008) indicated that stiffness of implanted segments was reduced and ROM increased, while facet joint force varied from 7.7 to 95.3 N depending on the insertion location. In another study on “ball-and-socket” cervical disc prostheses, Rousseau et al. (2008) suggested that pressure on facet joints may increase from 15 to 86% by adjusting the center of rotation and that a posterior center of rotation with a large radius was most effective in lowering pressure. Ahn and DiAngelo used a computer simulation model to show that facet-joint force on implanted segments increased during extension from 38.1 to 691 N in normal segments (Ahn and DiAngelo, 2008). The results of this study indicated that increased pressure on facet joints after CADR might occur with all loads and in various forms and degrees, possibly because of intrinsic design flaws or improper positioning of devices. In conclusion, both CSDP error and Prestige LP FE models might change the force transfer path of motion segments in facet joints.

Similar to other *in vitro* experiments, biomechanical experiments still need to be verified by *in vivo* animal experiments, especially in large animals. The comparable kinematics of the lower cervical spine was one of the criteria used in selecting non-human primates as the animal model for CADR. The upright spine mechanical system of non-human primates is suitable for CADR research. It is absolutely a “worst-case” scenario with regard to evaluating the biomechanics and durability of a cervical prosthesis. Non-human primates are not braced or immobilized after surgery, and they rapidly ambulate and perform their natural gymnastics, trapeze utilization, and cage rocking within the first postoperative week. In this study, human-sized CSDP could not be used in the non-human primates at C_5_-C_6_. The disc space dimensions of non-human primates are more accommodating to the smaller human-sized prosthetic implants; therefore, we adapted the size of CSDP according to non-human primate cervical spine anatomy. CT radiographic assessment showed the CSDP remained very stable at the operative level. Based on CT radiographic analysis, there were no incidences of migration or subsidence. Furthermore, the CVF structure fusion phenomenon indicated that the CSDP shows biochemical stability because the implant of surface osseo-integration and vertebral fusion require a stable mechanical condition. There were no significant perioperative complications (i.e., no loosening, no osteolysis, and no translational instabilities).

In this experiment, we aimed to determine CSDP biomechanical patterns of the cervical spine to understand underlying biomechanics and how the CSDP load transfer pattern affected segmental motion. The research has some limitations. First, the FE analysis was computational, and certain assumptions were made during the study. The assumption of the bond upon contact condition for bone and implant is a limitation of this study. The bone-implant interface is much more complex, with relative motions and separations, such as that of CDP-structure bone. However, an appropriately validated model can still provide comparative results to guide orthopedic surgery. The implantation experiment using the CSDP in non-human primates also verified its biomechanical stability. Second, the experimental period of CSDP implantation in non-human primates was short. CADR complications often take a long time to appear. Third, the friction-wear test simplifies the experimental conditions, such as friction movement and loading conditions. Simplified friction movement and loading conditions may not completely reflect the actual wear process of CSDP in the body. Despite these limitations, this research still provides sufficient information to understand more about CSDP biomechanics.

## Conclusion

This research has deepened the understanding of how the CSDP affects implant segmental motion as well as stress distribution in the bone-implant interface. Overall, it helps to understand the possible mechanism for the failures and how CSDP designs predispose to the problem. In the FE analysis, compared with those of the Prestige LP FE model, the biomechanical parameters of the CSDP FE model were relatively close to those of the intact FE cervical spine model. The CSDP error FE models proved that the performance of the CSDP, namely, ROM, bone-implant interface stress, and facet-joint force, is affected by the implantation position. In addition, we conducted friction-wear tests on the CSDP based on the results of the FE analysis to understand its degree of durability. Finally, the CSDP had satisfactory performance in non-human primate experiments.

## Data Availability Statement

The original contributions presented in the study are included in the article/supplementary material, further inquiries can be directed to the corresponding author/s.

## Ethics Statement

The animal study was reviewed and approved by Huazhen Biotechnology, Guangzhou, China.

## Author Contributions

ZLi, GS, and YH designed the experiments. JWo, ZLv, and JWa performed the research. YL assisted with the FE models and data analysis. KS and HZ contributed to animal study design. JWo and JWa prepared the manuscript. ZLi and GS revised the manuscript. All authors contributed to the article and approved the submitted version.

## Conflict of Interest

The authors declare that the research was conducted in the absence of any commercial or financial relationships that could be construed as a potential conflict of interest.
